# Circadian rhythm of daytime sleepiness in pediatric narcolepsy: A pilot study

**DOI:** 10.1002/brb3.3109

**Published:** 2023-06-07

**Authors:** Hui Ouyang, Zechen Zhou, Xiaotong Dai, Jun Zhang

**Affiliations:** ^1^ Department of Clinical Neurology People's Hospital of Peking University Beijing China; ^2^ Department of Peking University Health Science Center Beijing China

**Keywords:** circadian rhythms, EDS, narcolepsy pediatrics, questionnaires

## Abstract

**Background and objective:**

Excessive daytime sleepiness (EDS) has been far back reported as the most disabling symptom in the pediatric narcoleptic patients. However, there is a lack of studies to examine the circadian rhythms of EDS in pediatric narcoleptic population. Therefore, we aim to investigate the circadian rhythm of EDS in pediatric narcolepsy patients.

**Methods:**

We identified 50 pediatric narcoleptic patients (36 males and 14 females, mean age 13.68 ± 2.75 years). Data were collected through interviews and the relevant questionnaires (children depression inventory [CDI] and the pediatric quality of life inventory [PedsQL]).

**Result:**

The frequencies of sleep attacks during different intervals of the day differed significantly, with higher frequency in the morning (*p* < .001). The times of sleep attacks in the morning and in the afternoon were significantly associated with the degree of impairment on class and the severity of worry about sleepiness, with spearman correlation coefficient ranging from .289 to .496 (*p* < .05). The total scores of PedsQL and CDI differed significantly among morning sleepiness dominant, afternoon sleepiness dominant, and evening sleepiness dominant groups (*p* = .042, *p* = .040). The severity scores of the narcoleptic patients’ sleepiness had two peaks, one of which occurred at 16:00, and the other peaks occurred at about 11:00.

**Conclusion:**

These results suggest that changes based on the circadian rhythm of sleepiness of the pediatric narcoleptic patients should be made in the treatment strategy. In addition, regulating the secretion of melatonin could serve as a promising treatment to relieve sleepiness in the future.

## Graphical abstract

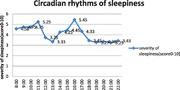

## 1 INTRODUCTION

Narcolepsy is a disabling sleep disorder with an estimated prevalence of 20–50 per 100,000 (Longstreth et al., 2007; Ohayon et al., 2002; Shin et al., 2008; Silber et al., 2002; Wing et al., 2002), the typical symptoms of which include excessive daytime sleepiness (EDS), cataplexy, hypnogogic hallucinations, and sleep paralysis, as well as abnormal regulation of the sleep–wake cycle (Dauvilliers et al., 2007; Dias Costa et al., 2014; Shelton & Malow, 2017). Symptoms of narcolepsy are often more severe in pediatric patients than adult patients (Plazzi et al., 2018). EDS, the hallmark of narcolepsy predisposes patients to serious performance decrements in multiple areas of function (Stores et al., 2006). It has been demonstrated that compared to age‐ and sex‐matched controls, children with narcolepsy have higher rates of behavioral, emotional, and educational problems (Cohen et al., 2018; Inocente et al., 2014; Stores et al., 2006), as well as lower life quality (Dodel et al., 2007; Jennum et al., 2012; Postiglione et al., 2018).

EDS with frequent lapses into sleep during daytime has been far back reported as the key symptom of narcolepsy (Postiglione et al., 2018; Sonka & Susta, 2012), it presents in 100% of patients with narcolepsy and is the most disabling symptom. Chronic sleepiness hinders pediatric narcoleptic patients socially and decreases quality of life (Inocente et al., 2014; Quaedackers et al., 2019; Zhang & Han, 2017). Meanwhile, EDS has been associated with decreased concentration and executive functioning compared to controls, some children with narcolepsy have clinically significant ADHD symptoms (Lecendreux et al., 2015; Stores et al., 2006), which may have great impact on the academic performance of the pediatric patients (Dhanju et al., 2018).

Despite the negative effect of EDS on children's academic achievement and extracurricular activities (Drake et al., 2003; Gibson et al., 2006), few researches have focused on the circadian rhythm of the EDS in pediatric patients with narcolepsy, as well as the impact of sleepiness at different times of the day on the life of pediatric narcoleptic patients. Recently, several studies have highlighted the importance of the endogenous circadian rhythms of narcolepsy patients (Blazejova et al., 2008; Donjacour et al., 2012; Filardi et al., 2016). However, there is a lack of studies to examine the circadian rhythms of narcolepsy symptoms in pediatric narcoleptic population.

Hence, the main purpose of the current study is to investigate the circadian rhythm of EDS in pediatric narcolepsy patients. The secondary aim of the study is to study the impact of sleepiness during different intervals of the day on the daily life of pediatric narcoleptic patients.

## 2 METHODS

### 2.1 Subjects and procedure

The study included 50 premedication patients, children, and adolescents (36 males and 14 females, mean age 13.68 ± 2.75 years) with a final diagnosis of narcolepsy evaluated at the sleeping center of Peking University, People's Hospital from May 2018 to March 2019. The circadian rhythms of 65 healthy adolescents were also evaluated (39 males and 24 females, mean age 14.56 ± 3.51 years). All patients fulfilled the current International Classification of Sleep Disorders, Third Edition.

Clinical evaluation was systematically conducted by the same sleep specialists. First, a structured questionnaire was administered to all participants to record demographic variables and medical history. Second, the clinical manifestations, especially EDS, were carefully inquired. The patients were asked to describe the subjective feeling, such as the severity of sleepiness and the impact of sleepiness on their life using the score 0–10. At last, the patients were asked to finish the children depression inventory (CDI) and the pediatric quality of life inventory (PedsQL) with the help of their parents and sleep specialists.

The study was approved by the Institutional Review Board of PHPU, and our protocols were performed in accordance with the Declaration of Helsinki regarding ethical guidelines for medical research involving human subjects, and written informed consent was signed by parents of children.

### 2.2 Statistical analysis

All continuous and categorical data were explored with descriptive (mean ± standard deviation) and frequency statistics for each group. Differences between groups in demographical data and scale scores were analyzed with ANOVA test. The relationship between clinical data and questionnaire scores as well as the test–retest reliability were explored with spearman correlation coefficient analyses.

Statistical analyses were conducted using SPSS 22.0 (SPSS, Inc. Chicago, IL, USA). Results with *p* < .05 were considered statistically significant.

## 3 RESULTS

### 3.1 Demographic, clinical, and neurophysiological characteristics

Fifty pediatric narcoleptic patients were identified, and their guardians were agreed to participate in the study. There were no significant differences in geographic location, gender, or race between participants and nonparticipants. The average age of patients who participate in the study was 13.68 years. The narcolepsy patients have more sleep attacks in the morning, with an average number of 1.95 times, and they have less sleep attacks in the evening, with an average number of 0.74 times. The mean scores of PedsQL 4.0 and CDI for the sample of children and adolescents with narcolepsy were qualitatively higher than the norm and they feel that sleepiness has an impact on their daily life, especially attending class. These results are presented in Table 1.

**TABLE 1:** Demographic and scores of subjective feelings and questionnaires (*n* = 50).

	Mean (SD)
Age	13.68 (2.75)
Times of sleep attacks in the morning (8:00–12:00)	1.95 (1.21)
Times of sleep attacks in the afternoon (13:00–18:00)	1.26 (0.89)
Times of sleep attacks in the evening (19:00–22:00)	0.74 (0.86)
Impact on class	6.62 (3.02)
Impact on outdoor activities	3.00 (2.58)
Impact on social communication	3.24 (3.11)
Worry about sleepiness	4.02 (2.90)
PedsQL 4.0	31.10 (16.41)
CDI	15.75 (7.37)
Percent male	72%

Abbreviations: CDI, children's depression inventory; PedsQL, pediatric quality of life inventory.

### 3.2 Reproducibility

Four of the narcoleptic patients were asked to describe the severity of sleepiness the second time after a median interval of 30 days (range: 15–45 days). The investigators and test environments were exactly the same as in the patients’ first completion of the questionnaire. All of the patients that participated in the test–retest described their symptoms as stable and unchanged between the first and second measurements. In terms of the test–retest reliability of the score of sleepiness, the reliability coefficient (intra‐class correlation coefficients) was .798 (95% CI: .683–.874, *p* < .001).

### 3.3 Differences in sleep attacks in the morning, afternoon, and evening

**FIGURE 1:**
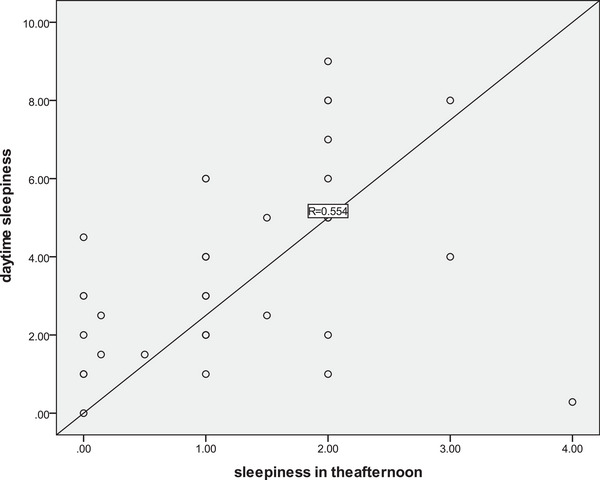
Correlation between times of sleep attack in the afternoon and sleep attack in the daytime.

As shown in Table 2, the frequencies of sleep attacks during different intervals of the day differed significantly, with *p*‐value less than .001. The times of sleep attacks in the morning and in the afternoon were significantly associated with the degree of impairment on class and the severity of worry about sleepiness, with spearman correlation coefficient ranging from .289 to .496 (*p* < .05). The times of sleep attacks in the morning, in the afternoon, and in the evening were all significantly associated with times of sleep attacks in the daytime. Among them, the times of sleep attacks in the afternoon were most closely associated with times of sleep attacks in the daytime (spearman correlation coefficient = .554, *p* < .001), and most closely associated the impairment on class attendance (spearman correlation coefficient = .438, *p* = .001). The results are presented in Table 2 and Figure 1.

**TABLE 2:** Differences in sleep attacks during different intervals of the day.

	Sleep attacks in the morning	Sleep attacks in the afternoon	Sleep attacks in the evening	*p*‐Value
Times of sleep attacks mean (SD)	1.95 (1.21)	1.26 (0.89)	0.74 (0.86)	<.001
Association with Impact on class^a^	0.390^b^	0.438^b^	0.166	
Association with Impact on outdoor activities^a^	0.222	0.099	0.216	
Association with impact on social communication^a^	0.053	−0.079	0.270	
Association with worry about sleepiness	0.496^b^	0.289^b^	0.035	

^a^ Spearman correlation coefficient.

^b^ *p* < .05.

### 3.4 Comparison of the life quality of narcoleptic patients with different sleepiness circadian rhythms

To study the effects of different circadian rhythms of sleepiness on the daily life of narcolepsy patients, we divided the narcolepsy patients into morning sleepiness dominant (have most frequent sleep attacks in the morning), afternoon sleepiness dominant (have most frequent sleep attacks in the afternoon), and evening sleepiness dominant (have most frequent sleep attacks in the evening) groups and then compared their scores of PedsQL and CDI. The total scores of PedsQL and CDI differed significantly among morning sleepiness dominant, afternoon sleepiness dominant, and evening sleepiness dominant patient (*p* = .042, *p* = .040). The results are presented in Table 3.

**TABLE 3:** The impact on life quality of patients with different sleepiness circadian rhythms.

	Morning sleepiness dominant	Afternoon sleepiness dominant	Evening sleepiness dominant	*p*‐Value
PedsQL	13.54 (2.61)	18.80 (5.95)	18.99 (8.49)	.042
CDI	14.03 (6.72)	21.11 (8.57)	15.25 (2.50)	.040

Abbreviations: CDI, children's depression inventory; PedsQL, pediatric quality of life inventory.

### 3.5 Circadian rhythms of the severity of sleepiness

**FIGURE 2:**
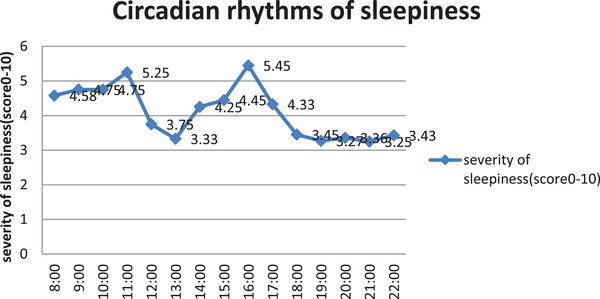
Circadian rhythms of sleepiness severity in pediatric narcolepsy patients. The blue spot represents the average severity of sleepiness at different time points (score 0–10, 0: not at all 10: extremely sleepy).

In order to study the circadian rhythms of the severity sleepiness of pediatric narcolepsy patients, we asked the patients to use the score 0–10 to describe their severity of sleepiness at different time points of the day (from 8:00 to 22:00). As shown in Figure 2, the severity scores of the narcoleptic patients’ sleepiness had two peaks, one of which occurred at 16:00, with an average score of 5.25. The other peaks occurred at about 11:00, with an average score of 5.45.

**FIGURE 3:**
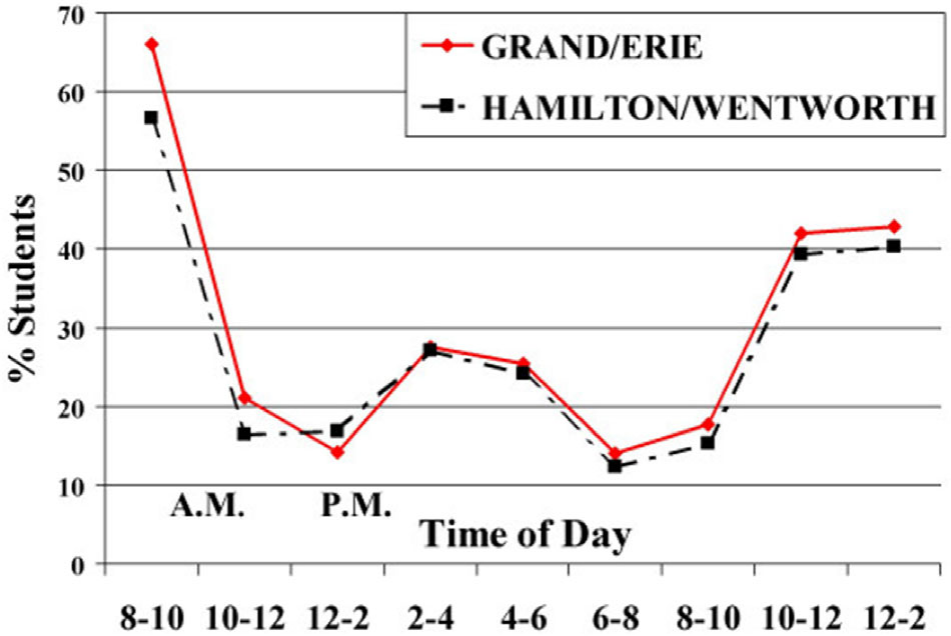
Circadian rhythms of sleepiness in normal students in Canada (Gibson et al., 2006).

**FIGURE 4:**
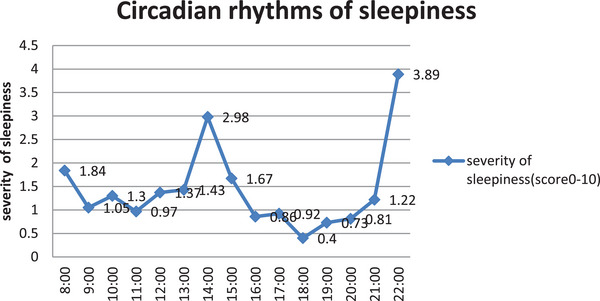
Circadian rhythms of sleepiness in normal students in China (*n* = 63).

## 4 DISCUSSION

Our study was the first in the world that specifically aimed at analyzing the circadian rhythms of sleepiness severity in a sizable group of pediatric narcoleptic patients. We found that the times of attacks during different intervals of the day were different. The severity of sleepiness at different time point showed a certain pattern circadian rhythm, with 2 peaks at 16:00 and 11:00, which is in consistent with the rhythm of melatonin secretion in narcolepsy patients to some degree (Donjacour et al., 2012). The circadian pattern of sleepiness in pediatric narcoleptic patients was different from the normal adolescent students (Gibson et al., 2006) (shown in Figures 2, 3, 4). The circadian rhythm of sleepiness in Chinese teenagers is generally consistent with that of Western teenagers reported in the literature, but there are slight differences. The peak in the severity of sleepiness among Chinese adolescents at 13:00 is significantly higher than that among Western adolescents, which may be related to the lifestyle habits of taking a nap at noon in Chinese adolescents.

Several studies have investigated the circadian rhythms in narcoleptic patients and demonstrated that particular circadian rhythmicity existed in narcoleptic patients, including the rhythm of psychophysiological performances, the rhythm of rest‐activity, and rhythm of melatonin secretion (Donjacour et al., 2012; Filardi et al., 2016; Schneider et al., 2004). In line with our findings, a previous study compared the circadian pattern of melatonin secretion of adult narcolepsy type 1 (NT1) patients and controls and showed that, although average hormone concentrations across the 24‐h did not differ between groups, the circadian pattern of melatonin release was altered in NT1, with patients presenting a higher proportion of melatonin secreted during daytime and a major peak of secretion in the early afternoon between 14:00 and 16:00 (Donjacour et al., 2012). Our results showed that the severity of sleepiness of the patients reached a peak at 16:00, this is consistent with the rhythm of melatonin secretion. This suggests that the rhythm of sleepiness did exist objectively, which may be associated with the melatonin secretion rhythm.

Current evidence indicates that low hypocretin‐1 level caused by orexin cell loss leads to sleepiness by destabilizing the ability of the circuits that initiate and sustain normal levels arousal and motor activity (Pintwala & Peever, 2017; Zhang & Han, 2017). However, the results of this study indicated that the EDS of pediatric narcoleptic patients might be not only associated with the hypocretin‐1 deficiency but also associated with the disturbed circadian control of melatonin release. Melatonin is a potential modulator of sleep, high levels of which have been associated with sleepiness (Donjacour et al., 2012; Reiter et al., 2010). Our results showed that the severity of sleepiness reached a peak at 16:00, this is somewhat consistent with the circadian rhythm of melatonin secretion. This suggests that the rhythm of sleepiness does exist objectively, which may be related to the altered rhythm of melatonin secretion.

Based on clinical experiences, sleep episodes happen more often in the morning and less frequently in the afternoon (Zhang & Han, 2017). According to our results, although the times of sleep attacks in the morning are the most frequent of the day, the sleepiness in the afternoon, however, also plays an important role in the life quality and subjective feeling of pediatric narcoleptic patients. Therefore, it is necessary to pay attention to the afternoon sleepiness in pediatric narcoleptic patients and control the afternoon sleepiness with appropriate drugs. The afternoon sleepiness is more closely associated with the frequency of daytime sleepiness, the CDI score and the subjective feeling about sleepiness, compared with sleepiness attacks during other intervals of the day. Therefore, it is crucial to control afternoon sleepiness in order to improve the life quality and the subjective feelings of pediatric narcoleptic patients.

The results indicated that some changes should be made in the narcolepsy treatment strategy. Recent medications have greatly improved the symptoms and wellbeing of young patients (Michel, 2013). However, the current drugs were usually given to narcolepsy patients once to twice per day, in the morning or at lunch (Evangelista et al., 2018; Kallweit & Bassetti, 2017). Excessive low dosage of drugs can lead to unsatisfied improve of symptoms, whereas high dosage of drugs is easy to cause side effects, such as tachycardia, anorexia, and insomnia. If we determine the time and dose of drugs administration based on the circadian rhythm of sleepiness of the pediatric narcoleptic patients, the appropriate therapeutic strategy can relieve the narcoleptic symptom, improve the life quality of narcoleptic patients, and avoid the side effects of drugs at the same time. In addition, regulating the secretion of melatonin could serve as a promising new kind of treatment to relieve sleepiness in pediatric narcoleptic patients in the future.

There are several limitations that need to be considered in the design of further studies. The sample size limits the generalizability of the results to a broader narcoleptic population to some degree. In addition, several of the negative findings may have been affected by our relative small sample size. Furthermore, the data were collected at a single study center. However, our institution receives patients from all regions of China. In the future, a study with larger sample size and possibly conducted by multiple‐centers is warranted and will allow us to confirm our results and possibly reveal more characteristics of the circadian rhythm of the EDS in pediatric narcoleptic patients.

## 5 CONCLUSION

In summary, we found the daytime sleepiness in pediatric narcoleptic patients presented a certain circadian rhythm, which was in consistent with the rhythm of melatonin secretion in narcoleptic patients. The severity of sleepiness in the afternoon was more closely associated the pediatric narcoleptic patients’ daily life and their subjective feeling about sleepiness. These findings may shed light on the treatment strategy based on the circadian rhythm of sleepiness and melatonin secretion in pediatric narcoleptic patients.

## CONFLICT OF INTEREST STATEMENT

The authors declare no conflict of interest.

### PEER REVIEW

The peer review history for this article is available at https://publons.com/publon/10.1002/brb3.3109.

## Data Availability

Authors declare that data and materials described in the manuscript are freely available to any scientist wishing to use them, without breaching participant confidentiality. The contact should be made via the corresponding author happyluckyfish@163.com.
